# Multi-Source Fatigue Fracture of 2Cr13 Martensitic Stainless Steel Compressor Blades: The Critical Role of Surface Integrity in Marine Engineering Reliability

**DOI:** 10.3390/ma19183965

**Published:** 2026-09-18

**Authors:** Yingwei Gao, Haoxian Dong, Chuan Lv, Yan Li, Lvjun Zhou, Yuze Song

**Affiliations:** 1 National Ocean Technology Center, Tianjin 300112, China; 2 Key Laboratory of Ocean Observation Technology, Ministry of National Resources, Tianjin 300112, China; 3 Space Star Technology Co., Ltd Tianjin Branch, Tianjin 300000, China; donghaoxian@163.com; 4 School of New Energy Engineering, Chengdu Technological University, Yibin 644000, China; lchuan1@cdtu.edu.cn; 5 School of Advanced Manufacturing, Sun Yat-Sen University, Shenzhen 518107, China

**Keywords:** marine engineering, compressor blades, 2Cr13 martensitic stainless steel, multi-source fatigue, surface integrity, failure analysis

## Abstract

Compressor rotor blades in marine engineering applications are exposed to harsh, corrosive environments and complex aerodynamic loads, making them prone to premature failure. This study investigates the fracture of 12th-stage 2Cr13 martensitic stainless-steel blades following a maintenance overhaul. Despite the replacement of several cracked blades, five blades fractured shortly after restart, accompanied by abnormal vibration. A comprehensive failure analysis was conducted, including macroscopic inspection, fractographic observation, energy-dispersive spectroscopy, metallographic examination, and mechanical property testing. The results indicate that the fractures are multi-source high-cycle fatigue. Crack initiation in the new blade originated from pre-existing transverse mechanical damage, while in the old blades, it initiated from sharp pits and microcracks introduced by sandblasting, which compromised surface integrity. The material exhibited a normal tempered sorbite structure and adequate mechanical properties, with slight strengthening due to service-induced precipitation and dislocation accumulation. The failure followed a typical evolution of multi-source initiation, propagation, crack coalescence, and final overload ductile fracture. These findings highlight the critical role of surface integrity in blade reliability.

## 1. Introduction

In marine engineering applications, the high-pressure air compressor system of an ocean monitoring vessel faces extremely harsh service conditions, characterized by high humidity, a chloride-laden atmosphere, and complex aerodynamic loads [1,2,3,4]. As a key component of gas turbines, compressor blades generally suffer from fatigue damage: the blades operate under varying temperatures and are subjected to substantial centrifugal forces induced by high rotational speeds, while dynamic forces arising from vibration further lead to the initiation and propagation of fatigue cracks [5,6,7]. Ensuring the long-term reliability of these components is crucial for the safe and efficient operation of marine energy and propulsion systems. However, despite advances in material design and surface protection, premature blade fractures still occur frequently and are often associated with improper maintenance operations, which compromise surface integrity and induce stress concentrations.

Generally, the failure of compressor components is mainly caused by fatigue, wear, surge, stall, and high vibration. Fatigue failure of compressor blades includes crack initiation, propagation, and final fracture, with the root cause being cyclic stress concentration following initial cracking, which may be induced by foreign object damage, corrosion, friction, or geometric discontinuities such as notches [8,9,10]. In addition, microstructural factors, such as precipitates, inclusions, and grain size inhomogeneity, can also play a significant role [11]. For example, the study by Kwon et al. [12] showed that coarse grains resulting from heat treatment and manufacturing processes promote fatigue cracking, thereby leading to premature failure. Previous studies have demonstrated that grit blasting, while effective for surface cleaning, can introduce surface defects such as embedded abrasive particles, increased roughness, and microcracks, which act as stress concentrators and promote fatigue crack initiation. For martensitic stainless steels, the fatigue behavior is particularly sensitive to surface condition, as the presence of surface irregularities can significantly reduce the fatigue endurance limit. Moreover, because compressor blades and vanes are in direct contact with air, environmental pollution significantly influences their degradation. One common form of corrosion in such environments is pitting corrosion, which may occur on blade surfaces exposed to chloride-containing salts [13]. Through numerical simulation and experiments, Mollapour et al. [14] investigated the pitting corrosion on the surface of a compressor blade made of C450 martensitic stainless steel and found that pits significantly increase local stress, thereby enhancing the tendency for crack initiation—a critical stage for fatigue fracture. This type of deterioration is known as corrosion fatigue, which has been previously reported in both AISI 410 stainless steel steam turbine blades (containing 12% Cr) and C450 stainless steel compressor blades (containing 15.7% Cr) [15]. Due to the combined effect of mechanical loading and chemical attack (corrosion), the fatigue endurance limit in a corrosive environment is significantly lower than that under non-corrosive conditions [16,17,18]. Nevertheless, research on fatigue damage of compressor blades under actual service conditions (especially after maintenance operations) for ocean monitoring vessels remains relatively scarce.

The present study conducts a systematic analysis of the fracture incident that occurred in the 12th-stage 2Cr13 martensitic stainless steel blades of a high-pressure air compressor on an ocean monitoring vessel following an overhaul. Although several cracked blades were replaced during the maintenance, five blades fractured shortly after restart, accompanied by abnormal vibration. To address this issue, a comprehensive set of methods was employed, including macroscopic damage inspection, fractographic observation (SEM), energy-dispersive spectroscopy (EDS), metallographic examination, and mechanical property testing (hardness and tensile tests). The aim of the study is to reveal the fracture nature and root causes of the blades. Hardness and tensile measurements were conducted at equivalent sampling locations on both the new and old blades, with at least five indentations per location for hardness testing and three specimens per condition for tensile testing, and all results are reported as mean ± standard deviation. Through a comparison of the surface condition, microstructure, and mechanical properties between new and old blades, as well as an exploration of the effect of service-induced microstructural evolution on mechanical behavior, the study seeks to provide insights into the failure mechanism.

## 2. Experimental Details

**Figure 1:**
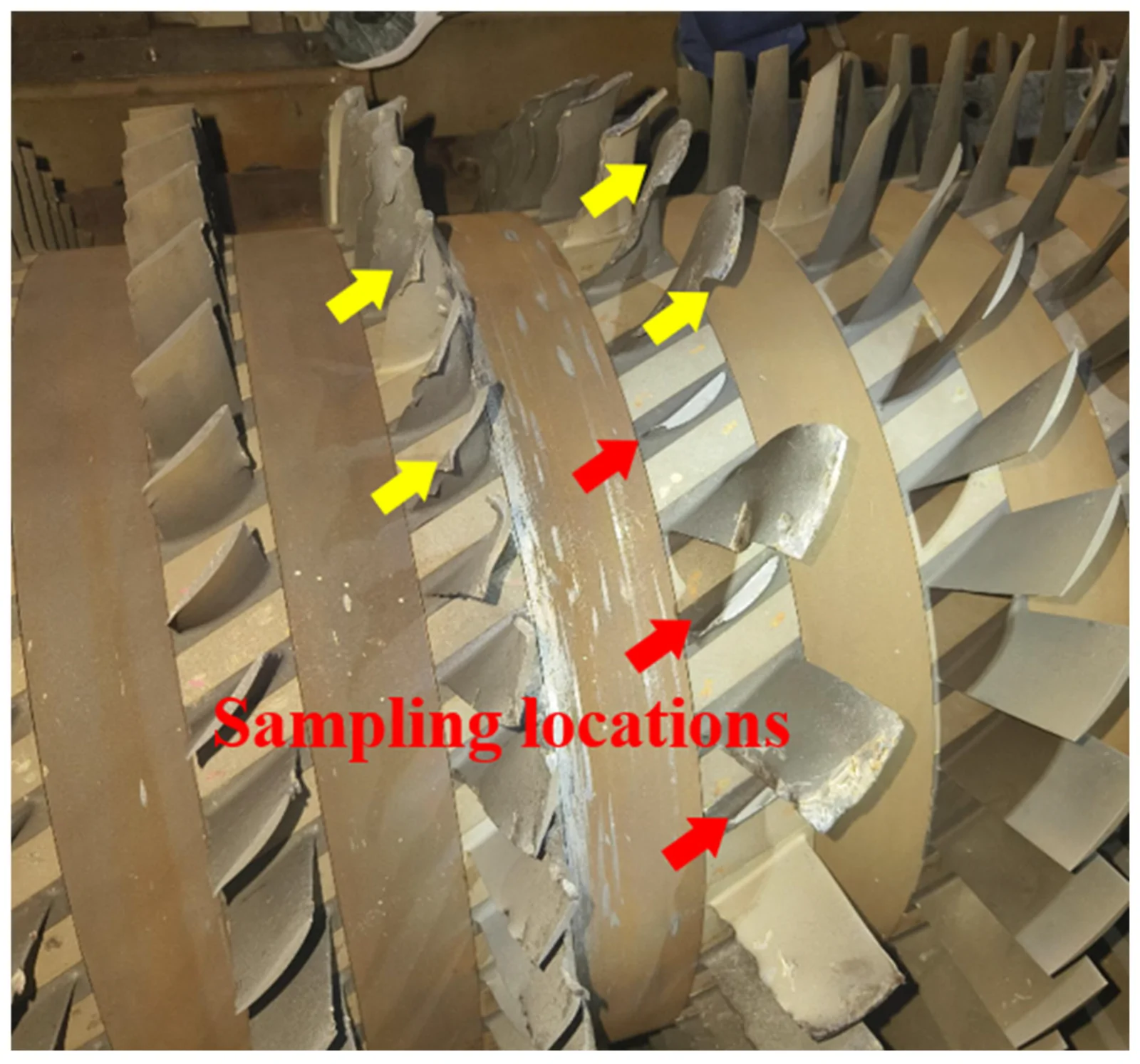
Macroscopic morphology of the damaged compressor impeller, showing fractured and severely deformed rotor blades.

The equipment is a compressor impeller (Figure 1) used in marine engineering, in which the fracture occurred in the 12th-stage compressor rotor blades. It is evident that the front-stage rotor blades show no damage, whereas both the same-stage and downstream-stage rotor blades exhibit varying degrees of impact damage. In addition, the impeller disk has undergone obvious circumferential fracture, with the red arrows indicating the crack and fracture locations, signifying a complete loss of structural integrity. Meanwhile, multiple blades display damage such as rolled edges, notches, and plastic deformation, as indicated by the yellow arrows. The complete 12th-stage impeller, consisting of 37 blades (4 newly replaced and 33 original with over two years of service), was investigated; among these, 5 fractured blades (1 new, 4 original) and 11 cracked blades were identified. All five fractured blades were subjected to macroscopic examination, and one new blade and one representative original blade were selected for detailed fractographic, microstructural, and mechanical property analyses.

**Figure 2:**
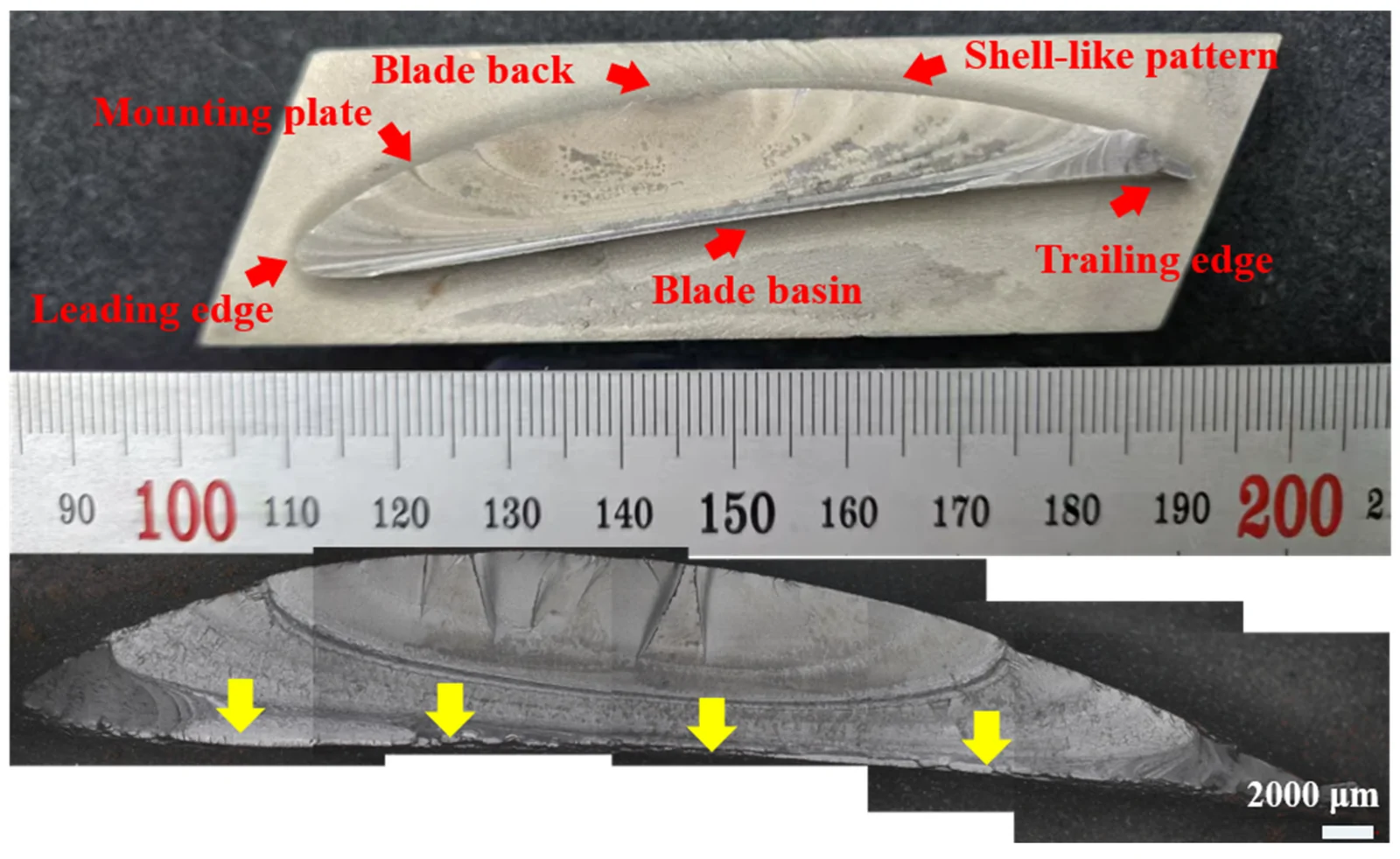
Optical microscopy (OM) image of the fracture region of the compressor impeller rotor blades.

As shown in Figure 2, the key structural features of the blade are indicated, including the leading edge, trailing edge, blade back, blade basin, and the characteristic shell-like pattern on the blade surface. The blade is attached to the source plate. To investigate the specific causes of damage, sampling was conducted in the marked regions. The lower portion presents a microscopic/fractographic image focusing on the fracture initiation zone, where the yellow arrows indicate the crack propagation path along the blade edge, and the red arrows pinpoint the precise location of crack initiation. To further explore the underlying fracture mechanisms, samples were collected from critical locations, including the fracture initiation zones, the regions exhibiting shell-like patterns, and the crack propagation paths of both new and old blades.

Sampling locations were selected from the fracture initiation zone, propagation zone, and final fracture zone on both the blade back and blade basin sides. Metallographic specimens were sectioned along the blade length direction using wire electrical discharge machining, mounted in conductive resin, ground with SiC papers up to 2000 grit, and polished with 1 μm diamond paste, followed by etching with 4% nital solution for approximately 10–15 s. For OM observation, an Olympus BX51M optical microscope was used at magnifications ranging from 50× to 500×. SEM examination was performed on fracture surfaces preserved in the as-fractured condition without any mechanical cutting, grinding, polishing, or ultrasonic cleaning to avoid altering characteristic fracture features. An FEI-ESEM Quanta 200 scanning electron microscope equipped with an EDS detector (Oxford Instruments) was employed at an accelerating voltage of 20 kV, with magnifications ranging from 50× to 10,000×. EDS analysis was conducted at multiple locations (Spot1–5) with a detection area of approximately 1 μm^2^ and an acquisition time of 60 s. Vickers hardness (HV0.1) and Brinell hardness (HBW) measurements were performed using a Wilson Wolpert 600MRD hardness tester (Wilson Wolpert, Norwood, MA, USA), with at least five indentations per location to obtain average values. Room-temperature tensile testing was conducted on an Instron 5967 universal testing machine at a crosshead speed of 2 mm/min, using specimens machined according to ASTM E8 standards [19], with three specimens tested for each condition to ensure statistical reliability.

## 3. Results and Discussion

### 3.1. Microstructure Morphology

**Figure 3:**
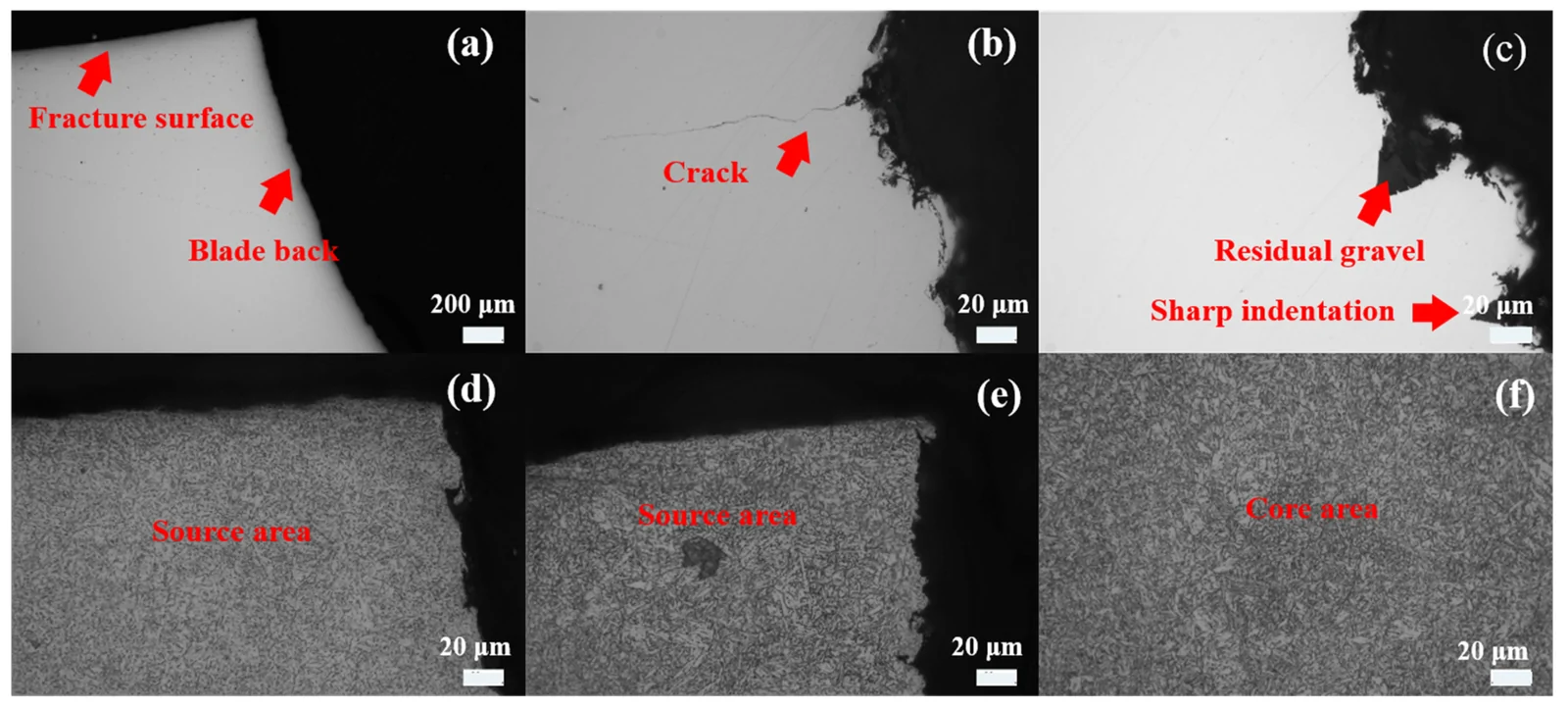
OM images of the fracture cross-section on the blade’s backside: (**a**,**d**) the fracture cross-section and surface morphology of the initiation zone of the new blade, respectively; (**b**,**c**) the fracture cross-section of the old blade; (**e**,**f**) the surface morphologies of the initiation zone and core region, respectively.

As shown in Figure 3, to compare the surface conditions of new and old blades and their potential influence on blade integrity, cross-sectional metallographic specimens were prepared along the blade length direction from the source region on the blade back. The surface morphologies of the new blade (Figure 3a) and the old blade (Figure 3b,c) exhibit significant differences. The new blade shows an uneven surface topography resulting from sandblasting, but no obvious microcracks or sharp pits are observed, indicating a relatively intact surface condition. In contrast, the old blade displays not only microcracks but also residual sandblasting grit and sharp pits in localized areas. Such sharp pits and microcracks are prone to act as stress concentrators during service, potentially initiating and propagating fatigue cracks under alternating loads, thereby posing a threat to the service reliability of the blades. As shown in Figure 3d–f, no obvious inclusions or other metallurgical defects are observed in the cross-sectional microstructures of either the new or the old blade, both of which exhibit uniform and homogeneous microstructures consisting of tempered sorbite.

### 3.2. Fracture Morphologies

**Figure 4:**
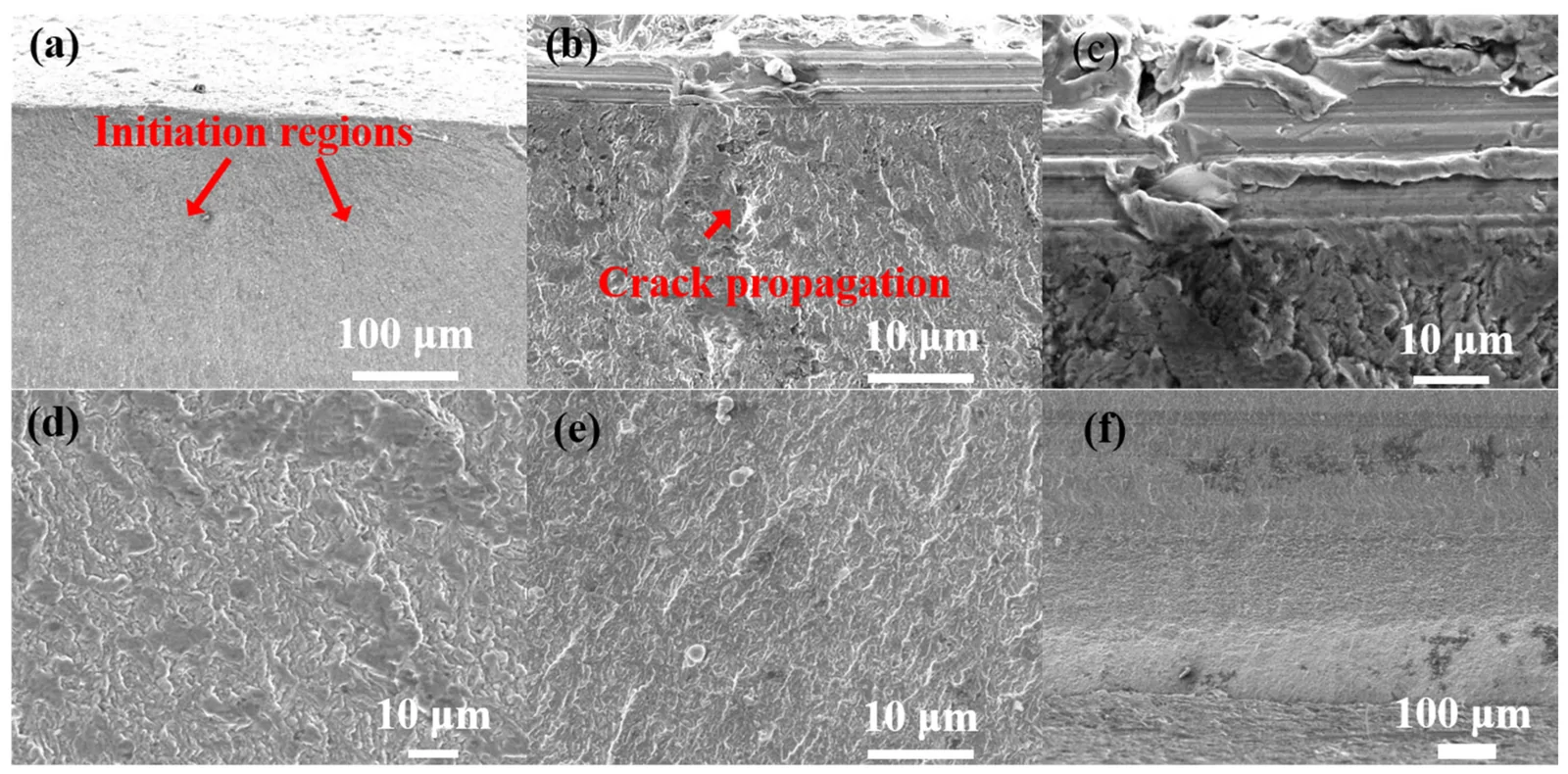
Fracture morphologies of the new blade: (**a**–**c**) initiation zone; (**d**) region near the initiation zone; (**e**) region away from the initiation zone; (**f**) final fracture zone.

Figure 4 illustrates the initiation mechanism and propagation process of the new blade fracture, as observed through microscopic examination of the fracture morphology using SEM. The results show that the fracture surface exhibits typical fatigue fracture characteristics and can be divided into the crack initiation zone, propagation zone, and final fracture zone. As shown in Figure 4a–c, the crack initiation zone is located at the bottom of the transverse mechanical damage on the blade surface near the blade back, displaying multiple initiation sites, indicating preferential crack initiation in this region. No obvious corrosion products or inclusions were observed in the initiation zone, suggesting that the fracture was not dominated by material defects or environmental corrosion. Notably, craters formed by sandblasting cover the surface of the transverse mechanical damage. Based on the overprint relationship, it is inferred that the transverse mechanical damage occurred prior to the sandblasting process, indicating that this damage was introduced during the original manufacturing or assembly process rather than during service. In Figure 4d,e, both the region adjacent to the initiation zone and the propagation zone farther away exhibit a cleavage morphology. Under higher magnification, fine fatigue striations can be observed, indicating that the crack underwent a well-developed stable propagation stage under alternating stress, characterized by a relatively low fatigue crack growth rate and a typical propagation process. Finally, the final fracture zone (Figure 4f) displays a dimple morphology, indicative of ductile overload fracture after the crack had propagated to a critical size, marking the ultimate instantaneous failure of the blade.

**Figure 5:**
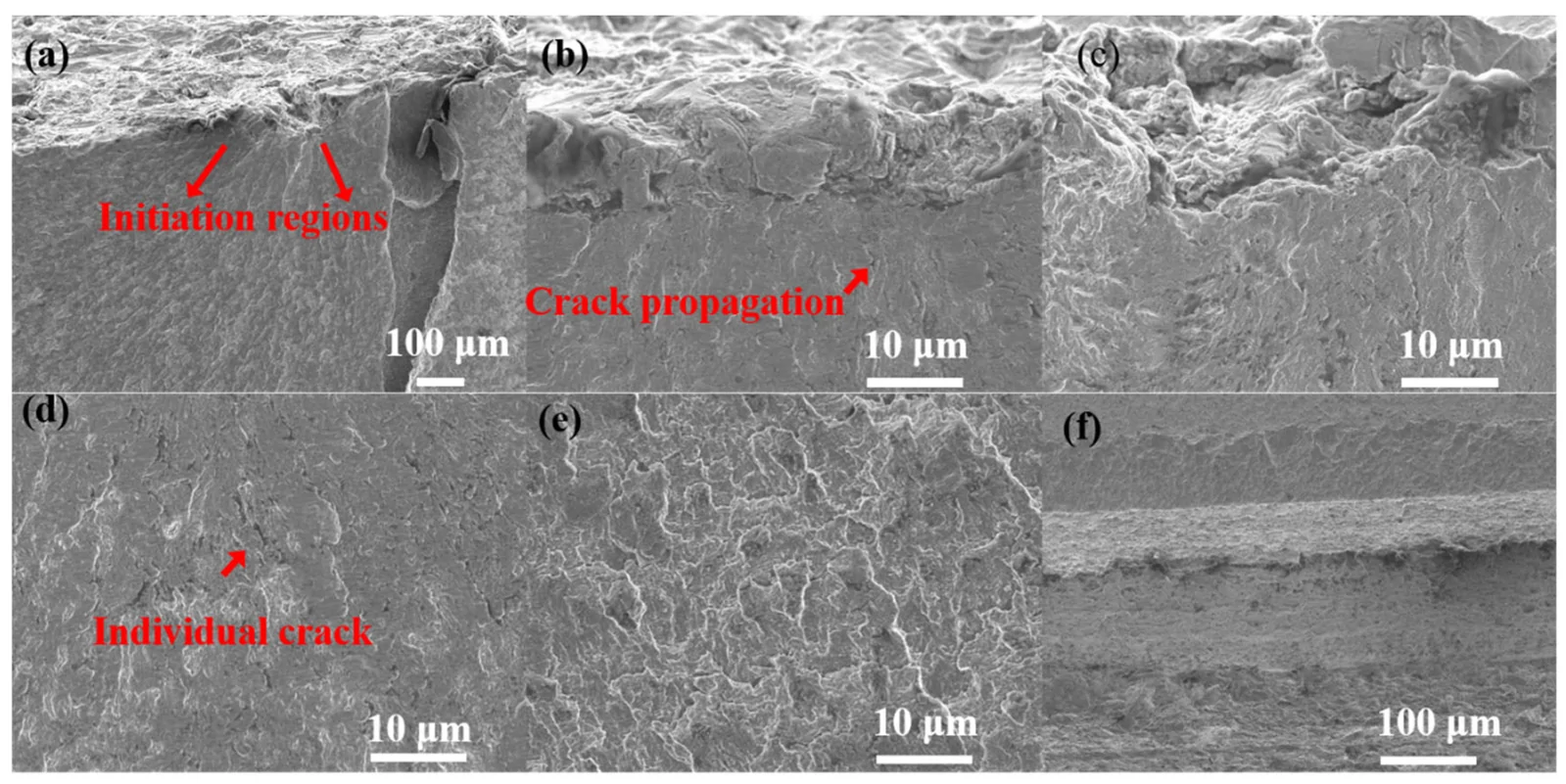
Fracture morphologies of the old blade: (**a**–**c**) initiation zone; (**d**) region near the initiation zone; (**e**) region away from the initiation zone; (**f**) final fracture zone.

In Figure 5a–c, the crack initiation zone of the old blade is located at the bottom of the pit formed by the sandblasting process near the blade back surface, exhibiting multiple point-source origins. No obvious corrosion products or non-metallic inclusions are observed in the initiation zone, indicating that crack initiation is not directly related to the intrinsic quality of the material. Notably, the initiation zone of the old blade corresponds precisely to the surface pits left by sandblasting, with sharp morphologies at the pit bottoms that are prone to act as stress concentrators during service, thereby becoming preferred sites for fatigue crack initiation. In Figure 5d–f, both the regions adjacent to the initiation zone and the propagation zones farther away display cleavage morphologies. Under higher magnification, fine fatigue striations can be observed, indicating that the cracks underwent a prolonged stable propagation stage under alternating stress, with fully developed fatigue propagation. Finally, in Figure 5f, the final fracture zone exhibits a dimple morphology, indicative of ductile overload fracture after the crack had propagated to a critical size. The fracture of the old blade initiated at the bottom of the surface pits formed by the sandblasting process and eventually fractured after fatigue propagation under alternating loads. A comparative analysis of the fracture characteristics of the new and old blades further corroborates the influence of the sandblasting process on blade surface integrity: if the sandblasting process is not properly controlled, the retained sharp pits can become potential sources of fatigue cracks, posing a significant threat to the service reliability of the blades [20,21,22].

**Figure 6:**
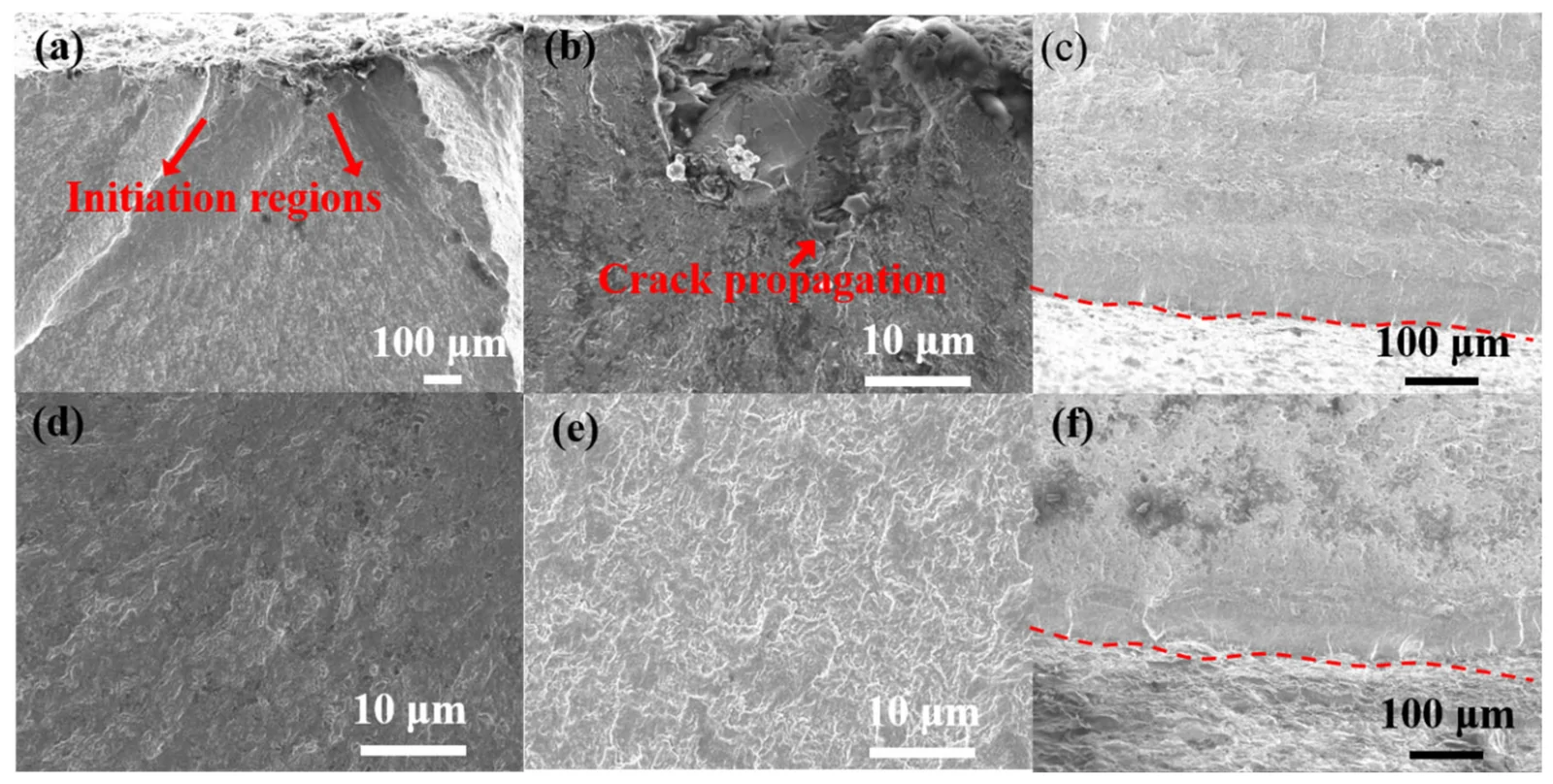
Fracture characteristics of the old blade: (**a**,**b**) typical initiation zones on the blade’s backside; (**c**) typical initiation zone on the blade’s basin side; (**d**) region near the initiation zone on the blade’s backside; (**e**) region away from the initiation zone on the blade’s backside; (**f**) morphology of the final fracture zone.

As shown in Figure 6, the fracture morphology of the old blade exhibits typical fatigue fracture characteristics, with two independent crack initiation zones (Figure 6a–c) located on the blade back and blade basin, respectively. In Figure 6a,b, the initiation zone on the blade back is situated at the bottom of the pits formed by the sandblasting process near the blade surface, displaying multiple point-source origins. No obvious corrosion features or non-metallic inclusions are observed in the initiation zone, indicating that crack initiation is not related to the intrinsic material quality but is closely associated with the surface pits left by sandblasting—the sharp morphology at the pit bottoms readily induces stress concentration during service, making them preferred sites for fatigue crack initiation. In Figure 6c, the initiation zone on the blade basin is located on the blade basin surface, also exhibiting multiple point-source origins, with no obvious corrosion features or inclusions observed. Notably, the propagation zone corresponding to the basin-side initiation zone occupies a relatively small area, suggesting that the crack initiated at this site later and underwent limited propagation. In Figure 6d,e, both the regions adjacent to the initiation zones and the propagation zones farther away on the blade back and basin display cleavage morphologies. Under higher magnification, fine fatigue striations are visible, indicating that the cracks underwent a prolonged stable propagation stage under alternating stress, with fully developed fatigue propagation. In Figure 6f, the final fracture zone exhibits a dimple morphology, indicative of ductile overload fracture after the crack had propagated to a critical size.

Synthesizing the fractographic analysis, the fracture of the old blade initiated with the formation and propagation of a fatigue crack at the bottom of the sandblasting pit on the blade back. As the crack propagated to a certain extent, the stress distribution on the blade was altered, leading to the initiation of a secondary crack at the stress concentration region on the blade basin. The coalescence and further propagation of the two cracks ultimately resulted in overload fracture of the blade. This finding further underscores the critical influence of the sandblasting process on blade surface integrity: improper control of the sandblasting process can leave sharp pits that act as potential fatigue crack initiation sites, posing a significant threat to the service reliability of the blades.

### 3.3. EDS Analysis

**Figure 7:**
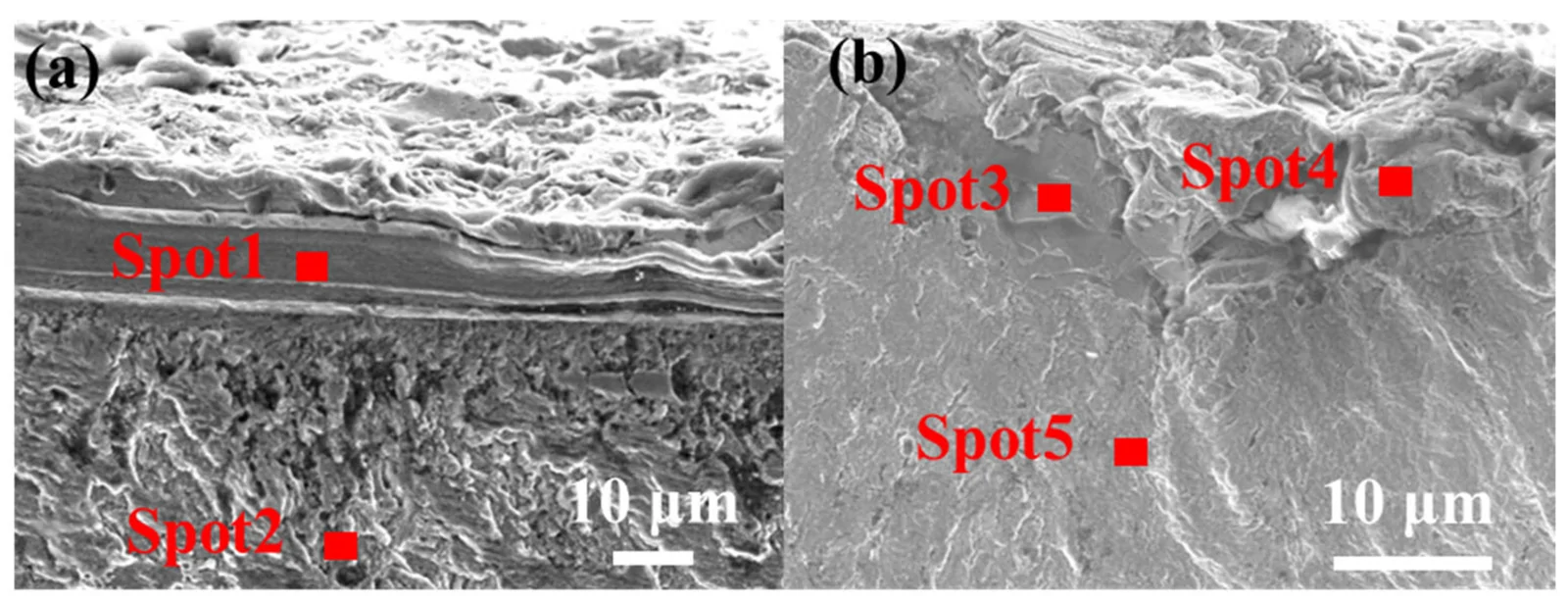
(**a**) Fracture region of the new blade; (**b**) fracture region of the old blade.

The results of SEM morphology observation and EDS quantitative analysis indicate significant differences in microstructure and elemental composition between the new blade and the old blade, reflecting material degradation behavior during service. The cross-section of the new blade (Figure 7a) exhibits a well-defined layered structure. The Spot1 region shows a dense and uniformly layered microstructure, with Fe and Cr contents of 74.9 wt.% and 11.9 wt.% (Table 1), respectively, and an oxygen content of only 1.6 wt.%. A relatively high carbon signal (11.1 wt.%) was detected in this region, which is attributed primarily to surface contamination from the mounting resin or residual organic compounds during specimen preparation, rather than to the intrinsic carbon content of the 2Cr13 steel matrix. This region is characteristic of the original matrix or protective coating with stable composition and intact structure. The Spot2 region displays a relatively rough and loose morphology with a few cracks, where the oxygen content (Table 1) increases to 4.3 wt.%, and the carbon content slightly decreases to 9.0 wt.%, suggesting minor initial oxidation or manufacturing-induced residual defects. The cross-section of the old blade (Figure 7b) exhibits pronounced service-induced damage characteristics. The Spot3 region shows a rough and loose morphology with a significantly elevated oxygen content of 8.6 wt.% (Table 1), while the Fe and Cr contents decrease to 64.7 wt.% and 10.8 wt.%, respectively. The detection of Si (2.2 wt.%) and trace Al (0.3 wt.%) in this region indicates the presence of residual blasting grit or corrosion products, accompanied by a significantly elevated oxygen content (8.6 wt.%), suggesting the formation of an oxygen-rich oxide scale that represents a primary degradation zone. The Spot4 region exhibits a torn and spalled porous morphology, which is inferred to be the exposed substrate following spallation of the oxide layer or protective coating. The elevated oxygen content (3.2 wt.%) at this location confirms oxidation of the exposed surface (Table 1). The Spot5 region serves as a representative of the post-service substrate, exhibiting a rougher morphology compared to the new blade, with an elevated oxygen content of 4.4 wt.% (Table 1) and a slightly reduced Fe content of 72.3 wt.%, reflecting the gradient oxidation and elemental depletion experienced by the substrate during service [23,24].

### 3.4. Hardness Testing

The Vickers hardness (HV0.1) and Brinell hardness (HBW) test results at different locations of the new and old blades are presented in Table 2. Significant differences in hardness level and distribution characteristics between the two blades reflect the influence of the service process on the mechanical properties of the material. For the new blade, the hardness at each location is generally low with minor fluctuations: the Vickers hardness ranges from 208 to 219, and the Brinell hardness ranges from 205 to 215. The hardness at the core and near the blade back surface is slightly higher than that at the initiation zone and near the fracture surface, with the lowest values recorded near the fracture surface (HV0.1 = 208, HBW = 205). This indicates a uniform microstructure with a slight positional gradient in mechanical properties. In contrast, the old blade exhibits significantly higher hardness at all tested locations, with a stable distribution: Vickers hardness ranges from 215 to 223, and Brinell hardness ranges from 215 to 220. The highest hardness is observed in the initiation zone (HV0.1 = 223, HBW = 220). Although the lowest hardness in the old blade is found near the fracture surface (HV0.1 = 215, HBW = 217), it remains higher than the corresponding locations in the new blade. These results indicate that high-temperature service led to a certain degree of strengthening of the blade matrix, which may be attributed to microstructural evolution such as precipitation phase formation, increased dislocation density, or elemental segregation. Moreover, the hardness distribution across different locations remains uniform, with no evidence of abrupt hardness reduction typically associated with severe degradation.

### 3.5. Room-Temperature Mechanical Properties

As shown in Table 3, the room-temperature tensile test results of the new blade and the old blade indicate significant differences in strength and ductility, reflecting the influence of high-temperature service on the mechanical behavior of the material. The new blade exhibits a tensile strength (Rm) of 689 MPa, a yield strength (Rp 0.2) of 457 MPa, and a percentage elongation after fracture of 27%. In comparison, the old blade shows a tensile strength of 683 MPa, a yield strength of 476 MPa, and a percentage elongation after fracture of 29%. Relative to the new blade, the old blade experiences a slight decrease in tensile strength (approximately 0.9%), remaining at a comparable level; a notable increase in yield strength (approximately 4.2%); and a slight increase in elongation (approximately 7.4%), indicating no degradation in ductility.

The macroscopic conchoidal markings and microscopic fatigue striations observed on the blade fracture surfaces indicate that the fracture of the blades is fatigue-related. The fatigue propagation zone occupies a large proportion of the fracture surface, and fine fatigue striations are observed in the propagation zones both near and away from the crack initiation sites, suggesting fully developed fatigue propagation. Therefore, the fracture nature of the blades can be identified as high-cycle fatigue. The blade microstructure and hardness are normal, the mechanical properties are satisfactory, and the chemical composition meets the required standards. Hence, the blade fracture is not related to material quality. On the new blade, transverse mechanical damage exists at the initiation zone on the blade back surface; on the old blade, pits formed during sandblasting cleaning are present. These features compromise the surface integrity and increase the stress concentration in these regions. However, under normal stress levels, such damage is insufficient to induce multi-source cracking, implying that the blades experienced relatively high stress levels during crack initiation.

## 4. Conclusions

Based on the findings from macroscopic damage characterization, microstructural analysis, energy-dispersive spectroscopy, mechanical property testing, and fractographic observation, the following conclusions can be drawn:

1. The blade fracture is characterized as a multi-source fatigue fracture. Fractographic examination revealed that the crack initiation region of the new blade is located at the bottom of the transverse mechanical damage on the blade body, while that of the old blade originates at the bottom of surface pits and at the stress concentration region on the blade’s basin side. Multiple initiation sites were observed in both cases, consistent with multi-source fatigue characteristics.
2. Surface integrity differs between the new and old blades examined. The blade back surface of the new blade showed no observable microcracks or sharp pits after sandblasting treatment, whereas the blade back surface of the old blade exhibited residual blasting grit, sharp pits, and microcracks. These features were identified as potential stress concentrators and preferential initiation sites for fatigue cracks in the old blade.
3. The fracture morphology of the old blade exhibits two distinct fatigue initiation zones, located on the blade’s back and basin sides. The propagation zone on the blade’s backside occupies a larger area than that on the blade’s basin side. The final fracture zone displays a dimple morphology, consistent with overload ductile failure. These observations support a failure process involving multiple crack initiations, propagation, and eventual coalescence leading to final fracture.

## Figures and Tables

**Table 1:** EDS analysis results of the initiation zone and adjacent regions (wt.%).

Position/Element		Fe	Cr	C	O	Si	Mn	Al
New Blade	Spot1	74.9	11.9	11.1	1.6	0.4	/	/
	Spot2	74.8	11.5	9.0	4.3	0.3	/	/
Old Blade	Spot3	64.7	10.8	12.8	8.6	2.2	/	0.3
	Spot4	77.3	12.6	6.2	3.2	0.6	/	/
	Spot5	72.3	11.1	11.6	4.4	0.6	/	/

**Table 2:** Test locations for the hardness of new and old blades.

Blade	New Blade				Old Blade			
Position	Initiation Zone	Core	Near Fracture Surface	Near Blade Back Surface	Initiation Zone	Core	Near Fracture Surface	Near Blade Back Surface
HV0.1	212 ± 5.6	217 ± 8.5	208 ± 3.6	219 ± 2.3	223 ± 6.2	220 ± 5.9	215 ± 5.4	225 ± 5.7
HBW	205 ± 4.5	210 ± 7.3	205 ± 4.2	215 ± 5.7	220 ± 6.5	215 ± 6.2	217 ± 4.9	220 ± 4.6

**Table 3:** Test results on the mechanical properties of new and old blades.

Sample	R_m_ (MPa)	R_p0.2_ (MPa)	Percentage Elongation After Fracture (%)
New Blade	689 ± 13	457 ± 8.5	27 ± 2.2
Old Blade	683 ± 12	476 ± 6.9	29 ± 1.3

## Data Availability

The original contributions presented in this study are included in the article. Further inquiries can be directed to the corresponding authors.
